# Immunohistochemical Predictors of Bone Metastases in Breast Cancer Patients

**DOI:** 10.1007/s12253-015-9957-0

**Published:** 2015-06-11

**Authors:** Piotr Winczura, Katarzyna Sosińska-Mielcarek, Renata Duchnowska, Andrzej Badzio, Joanna Lakomy, Hanna Majewska, Rafał Pęksa, Beata Pieczyńska, Barbara Radecka, Sylwia Dębska-Szmich, Krzysztof Adamowicz, Wojciech Biernat, Jacek Jassem

**Affiliations:** Medical University of Gdańsk, Gdańsk, Poland; Regional Oncology Center, Gdańsk, Poland; Regional Oncology Center, Opole, Poland; Military Institute of Medicine, Warsaw, Poland; Medical University of Łódź, Łódź, Poland; Radiotherapy Center Elbląg, ul. Królewiecka 146, 82-300 Elbląg, Poland

**Keywords:** Breast cancer, Bone metastases, Predictive factors, Immunohistochemistry

## Abstract

Bones are the most common metastatic site of relapse in breast cancer patients and the prediction of bone metastases (BM) risk might prompt developing preventive and therapeutic strategies. The aim of the study was to correlate imumohistochemical (IHC) expression of selected proteins in primary breast cancer with the occurrence of BM. We analyzed expression of proteins potentially associated with BM in primary tumors of 184 patients with metastatic breast cancer (113 with- and 71 without BM). Expression of estrogen receptor (ER) in primary tumor was more common in patients with- compared to those without BM (74 vs. 45 % respectively, *p* = 0.0001), whereas in this subset less common was expression of parathyroid hormone related protein receptor type 1 (16 vs. 34 %, respectively, *p* = 0.007) and cytoplasmic expression of osteopontin (OPNcyt; 1.9 vs. 14 %, respectively, *p* = 0.002). The relationship between expression of ER and OPNcyt and the occurrence of BM was confirmed in the multivariate analysis. The ER-positive/OPNcyt negative phenotype was significantly more common in patients with- compared to those without BM (75 and 25 %, *p* < 0.0001, respectively; HR 1.79, *p* = 0.013). Luminal A (43 vs. 23 % respectively, *p* = 0.009) and luminal B/HER2-positive (16 vs. 4.9 % respectively, *p* = 0.032) subtypes were more common in patients with- compared to those without BM, whereas triple negative breast cancer subtype was less common (16 vs. 38 %, *p* = 0.002).

## Introduction

Despite continuous progress in locoregional and systemic therapies, substantial number of breast cancer patients experience relapse. Bones constitute the most common metastatic site in advanced breast cancer, with the occurrence of up to 80 % [1, 2]. Patients with bone metastases (BM) have better prognosis than those with visceral involvement, and some may achieve long term survival. On the other hand, BM, due to pain and pathological fractures, significantly affect quality of life and remain a clinical challenge [1–3]. The development of BM in breast cancer is a complex phenomenon and includes dynamic interaction between malignant cells and bone tissue [4]. BM are typical for hormone receptor positive breast cancers [5–7] Results of randomized studies using adjuvant bisphosphonates to prevent BM in unselected groups of breast cancer patients have been inconsistent [8, 9]. Molecular markers facilitating individual risk assessment of BM might be therefore clinically useful.

The aim of our study was to correlate imumohistochemical (IHC) expression of selected proteins in primary breast cancer with the occurrence of BM. The subject of this analysis were four proteins routinely determined in breast cancer patients: estrogen receptor (ER), progesterone receptor (PgR), human epidermal growth factor receptor 2 (HER2) and Ki67, and six investigational proteins selected by their presumed association with increased risk of BM: cyclooxygenase 2 (COX2) [10], cytokeratins 5/6 (CK5/6) [5], chemokine receptor (CXCR4) [11], parathyroid hormone related protein receptor type 1 (PTHrPR1) [12, 13], osteopontin (OPN) [14, 15] and calcium sensing receptor (CaSR) [16].

## Materials and Methods

### Patient Population

The study was performed in 10 Polish oncology institutions and was approved by the Ethics Committee of the coordinating center, the Medical University in Gdańsk. The study group included 184 patients with metastatic breast cancer, including 113 patients with- and 71 without diagnosed BM (Table 1). The diagnosis of BM was based on specific symptoms confirmed by imaging (X-ray, CT, MRI or bone scintigraphy) or pathology. No screening for asymptomatic BM was performed. The group with BM included also other locations of metastatic disease (viscera, soft tissue). Notably, visceral metastases were present in over 90 % of patients in the non-BM group. Clinical data were extracted directly from the medical charts.

**Table 1:** Patient characteristics

Variable	Bone metastases group (*N* = 113)	Extraskeletal metastases group (*N* = 71)	Total (*N* = 184)
Mean age (range)	52 (30–78)	54 (30–83)	53 (30–83)
Menopausal status			
- Premenopausal	51 (45 %)	26 (37 %)	77 (42 %)
- Postmenopausal	62 (55 %)	42 (59 %)	104 (57 %)
- Unknown		3 (4.2 %)	3 (1.6 %)
Histology			
- Ductal	88 (78 %)	58 (82 %)	146 (79 %)
- Lobular	18 (16 %)	9 (13 %)	27 (15 %)
- Mixed (ductal/lobular)	3 (2.7 %)	3 (4.2 %)	6 (3.3 %)
- Other types	4 (3.5 %)	1 (1.4 %)	5 (2.7 %)
Stage at diagnosis			
- I	8 (7.1 %)	3 (4.3 %)	11 (6 %)
- II	51 (45 %)	32 (45 %)	83 (45 %)
- III	40 (35 %)	34 (49 %)	74 (41 %)
- IV	14 (12 %)	1 (1.4 %)	15 (8.2 %)
- Unknown	0 (0 %)	1 (1.4 %)	1 (0.5 %)
Primary surgery			
- Mastectomy or breast conserving surgery	99 (88 %)	68 (96 %)	167 (91 %)
- Biopsy only	14 (12 %)	3 (4.2 %)	17 (9.2 %)
Radiotherapy			
- Yes	100 (89 %)	47 (66 %)	147 (79 %)
- No/unknown	13 (11 %)	24 (34 %)	37 (21 %)
Chemotherapy			
- Yes	102 (91 %)	66 (93 %)	168 (91 %)
- No/unknown	11 (9.4 %)	5 (6.5 %)	16 (8.7 %)
Hormonal therapy			
- Yes	78 (69 %)	39 (55 %)	117 (63 %)
- No/unknown	35 (31 %)	32 (45 %)	67 (37 %)
Dominant site of metastases^a^			
- Bones	69 (61 %)	0 (0 %)	69 (38 %)
- Viscera	44 (39 %)	65 (91 %)	109 (59 %)
- Soft tissues	0 (0 %)	6 (8.5 %)	6 (3.5 %)

^a^Dominant site of metastases refers to the metastatic site with the worst prognosis

### Preparation of the Tissue Microarrays (TMA)

For each case, a pathologist identified representative tumor area on paraffin fixed block, using a stained hematoxylin-eosin section on a glass slide. From each block two tissue cores of 1.5 mm were taken. Manual Tissue Arrayer manufactured by Beecher Instruments (MTAI K7 Biosystems) was used for preparation of tissue microarrays.

### Protein Expression

Expression of each protein was evaluated on stained 4 μm sections. Characteristics of the antibodies used in the study are presented in Table 2. Primary antibodies were incubated according to manufacturer’s instructions and staining was performed with the use of Novolink Polymer Detection System by Novocastra.

**Table 2:** Antibodies used in the study

Protein	Manufacturer	Antigen retrieval	Incubation time	Dilution	Method of evaluation
ER	Novocastra,NCL-L-ER-6F11	HIER^a^ (ph6)	1.5 h	1/400	Semi-quantitative
PgR	Novocastra,NCL-L-PGR-312	HIER (ph6)	1.5 h	1/400	Semi-quantitative
HER2	Novocastra,NCL-L-CB11	HIER (ph9)	1.5 h	1/50	Semi-quantitative
Ki 67	Novocastra, NCL-L-Ki67-MM1	HIER (ph6)	1.5 h	1/1200	Semi-quantitative
OPN	Abcam, ab 33046	HIER (ph6)	1.5 h	1/200	Semi-quantitative
CXCR4	Invitrogen, 358800	HIER (ph6)	1.5 h	1/25	Semi-quantitative
CaSR	Pierce Bio., PA1-37213	HIER (ph6)	1.5 h	1/50	Semi-quantitative
COX2	Abcam, ab 10940	HIER (ph6)	1.5 h	1/100	Semi-quantitative
CK 5/6	Millipore, MAB1620	HIER (ph9)	1.5 h	1/400	Qualitative
PTHrPR1	Abcam, ab 3271	HIER (ph6)	1.5 h	1/50	Semi-quantitative

^a^HIER – heat induced epitope retrieval (in pressure cooker under 120 hPa for 2.5 min)

**Fig. 1:**
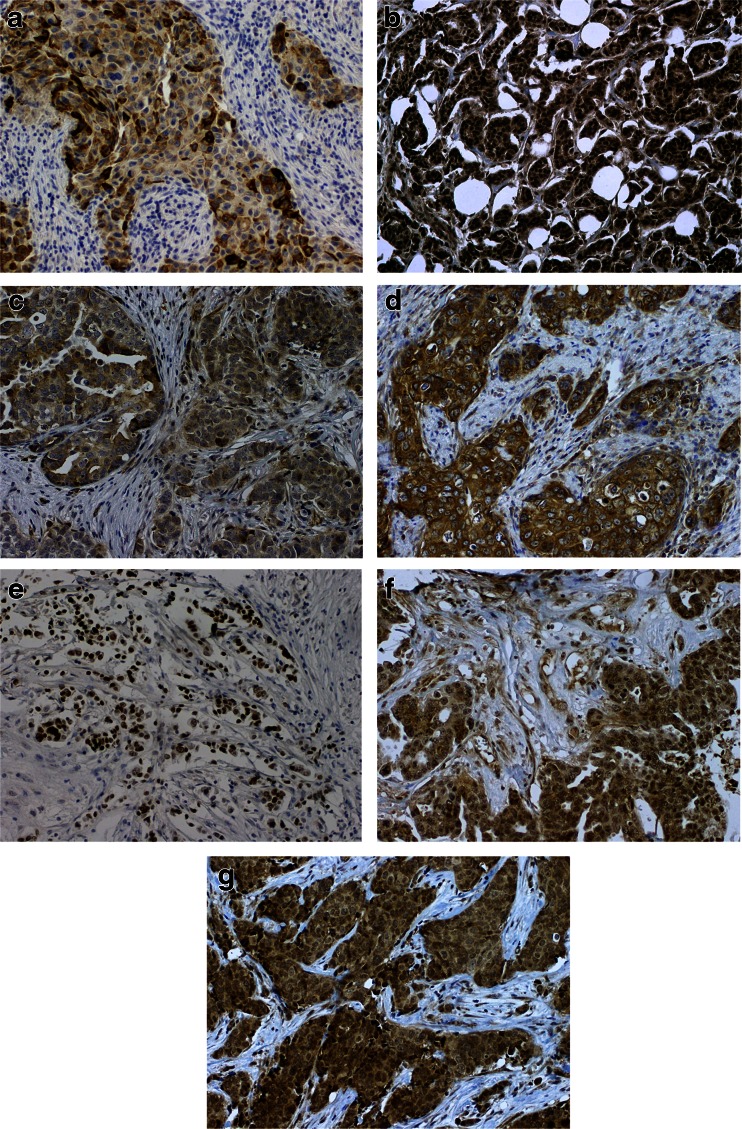
Representative images of particular proteins’ expression (magnification ×200). **a** CK 5/6, **b** CXCR4, **c** CaSR, **d** COX2, **e** OPN_n_, **f** OPN_cyt_, **g** PTHrPR1

For ER and PgR the staining was considered positive when at least 1 % of tumor nuclei expressed the proteins [17]. HER2 expression was classified as positive (3+) if strong membranous reaction was seen in more than 30 % of the invasive cancer cells. In the case of equivocal staining (2+) fluorescent in situ hybridization (FISH) data were taken from medical charts, and if not available, FISH was performed using TMA. FISH results were considered positive if HER2/chromosome 17 centromere signals ratio was higher than 2.2 [18]. Ki67 immunostaining was considered high if ≥ 14 % of the tumor nuclei stained positive [19, 20]. CK 5/6, tumors were classified as positive if any membranous or cytoplasmic staining was present [21]. Immunostaining for COX2 was considered positive when moderate or strong cytoplasmic expression was present in ≥ 10 % tumor cells [22]. CXCR4 expression was assessed separately in the nucleus (CXCR4_n_) and cytoplasm (CXCR4_cyt_), and staining in ≥ 1 % of the cells was considered positive [23]. CaSR was assessed in the cytoplasm [16] and immunostaining was considered positive if moderate to strong reaction was seen in ≥ 50 % of cancer cells. For OPN and PTHrPR1 both cytoplasmic intensity (0 - no staining, 1 - weak, 2 - moderate, 3 - strong) and percent of stained cells (1 for ≤1 %, 2 for >1–10 %, 3 for >10–33 %, 4 for >33–66 %, 5 for >66 %) were assessed and summarized, analogous to Allred score used for ER and PgR assessment. The cutoff values for PTHrPR1 and OPN_cyt_ were optimized at >6 and >7, respectively, to maximize the hazard risk of bone relapse between patients with expression levels above vs. below the cutoff. For OPN the nuclear expression was additionally assessed, and staining in ≥ 50 % of the cells was considered positive. Positive staining examples of CK 5/6, COX2, OPN, PTHrPR1, CaSR and CXCR4 are shown in Fig. 1.

Based on the expression of ER, PgR, HER2 and Ki67, five main breast cancer phenotypes were distinguished [19]. ER- and/or PgR-positive, HER2-negative and Ki67-low tumors were classified as luminal A, ER- and/or PgR-positive, HER2-negative and Ki67 high - as luminal B/HER2-negative, ER- and/or PgR-positive and HER2-positive - as luminal B/HER2-positive, ER/PgR-negative and HER2-positive - as nonluminal/HER2-positive, and ER-and/or PgR-negative and HER2-negative - as triple negative.

### Statistical Methods

STATA 8.0 software was used for statistical analyses. Categorical variables were compared using two-sided Pearson’s chi-square test, and for small group samples Fisher exact test was applied. Overall survival was estimated as time from initial pathological diagnosis to death and time to BM was the time from initial pathological diagnosis to confirmation of BM either with imaging or pathological examination. Overall survival and time to BM were calculated using Kaplan-Meier method and differences were analyzed with log-rank test. Statistical significance was considered at *p* < 0.05. To evaluate the risk of BM, Cox proportional hazards regression was used.

## Results

**Fig. 2:**
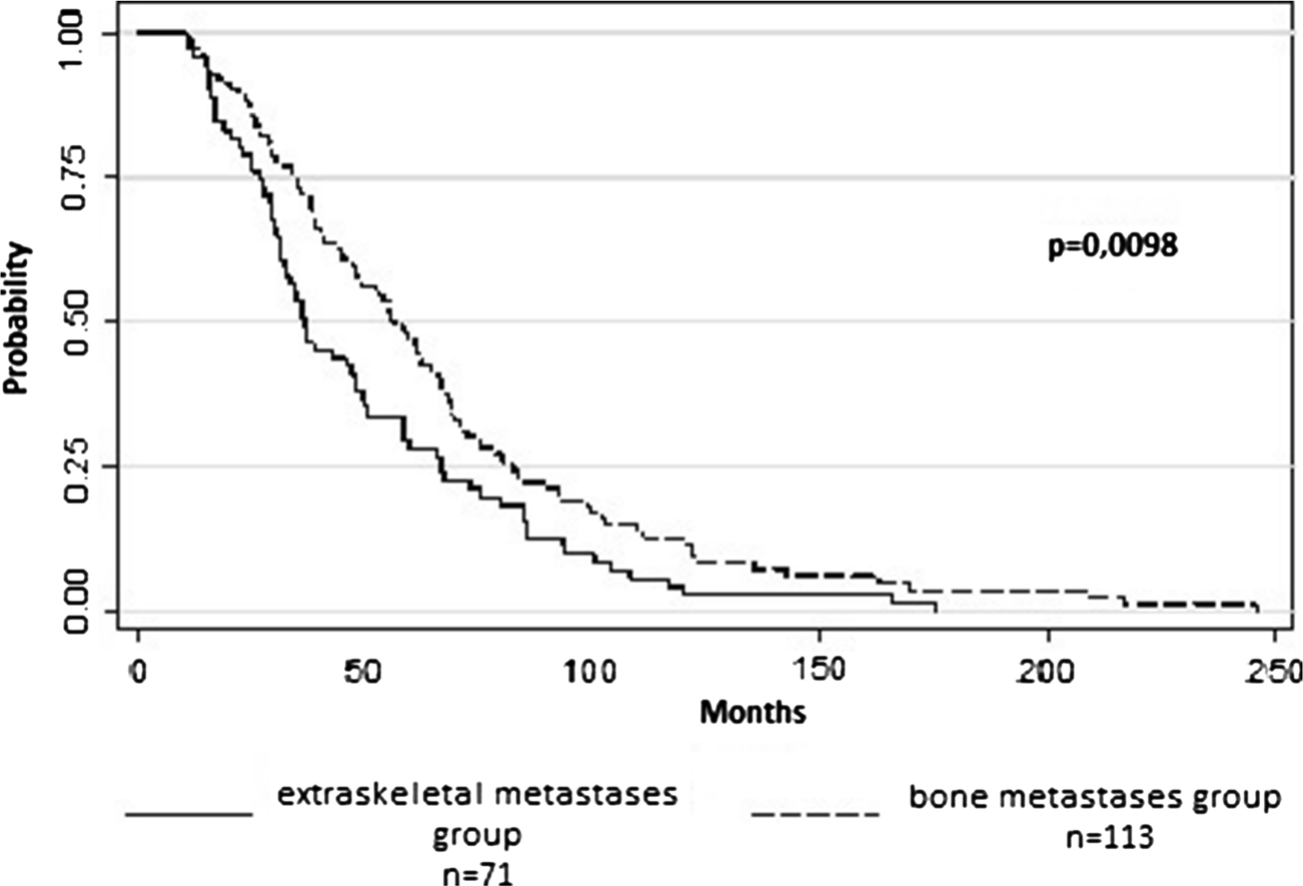
Overall survival of patients with bone metastases (*n* = 113) and with extraskeletal metastases (*n* = 71)

Median survival in the entire group was 48 months; 56 and 37 months in patients with- and without BM, respectively (log-rank; *p* = 0.0098, Fig. 2).

ER expression in primary tumor was present in 74 and 45 % of patients with- and without BM, respectively (*p* = 0.0001; Table 3). The respective figures for PgR were 55 and 42 % (*p* = 0.096). There were no significant differences in the occurrence of HER2 positivity (*p* = 0.252) and high Ki67 expression between both groups (*p* = 0.13). CaSR was overexpressed in most of the analyzed specimens; in 93 and 84 % of patients with- and without BM respectively (*p* = 0.053). OPN_cyt_ expression was less common in patients with- compared to those without BM (1.9 and 14 %, respectively, *p* = 0.002), whereas nuclear OPN (OPN_n_) expression did not differ significantly between both groups (37 and 48 %, respectively, *p* = 0.128). PTHrPR1 overexpression was around twice less common in patients with- compared to those without BM (16 and 34 %, respectively, *p* = 0.007). COX2 expression was a common occurrence in both groups (88 and 79 % in patients with- and without BM, respectively, *p* = 0.148). CXCR4_cyt_ was found in 87 and 90 % of patients with- and without BM, respectively (*p* = 0.74), and nuclear CXCR4_n_—in 64 and 66 %, respectively (*p* = 0.92). Expression of CK 5/6 was more common in triple negative breast cancer subtype compared to all other subtypes combined (45 and 12 %, respectively, *p* = 0.0001) and slightly less common in subjects with- compared to those without BM (18 and 30 %, respectively; *p* = 0.066).

**Table 3:** Expression of particular proteins in patients with- and without BM (univariate analysis; significant p values marked in bold)

Protein	Bone metastases group (%)	Extraskeletal metastases group n (%)	*p* value
ER	83 (74)	32 (45)	**0.0001**
PgR	62 (55)	29 (42)	0.096
HER2	25 (24)	11 (16)	0.252
Ki 67	36 (37)	31 (49)	0.13
CaSR	95 (93)	57 (84)	0.053
OPN_n_	38 (37)	31 (48)	0.128
OPN_cyt_	2 (1.9)	9 (14)	**0.002**
CXCR4_cyt_	91 (87)	60 (90)	0.74
CXCR4_n_	67 (64)	44 (66)	0.92
PTHrPR1	16 (16)	22 (34)	**0.007**
COX2	80 (79)	58 (88)	0.148
CK 5/6	19 (18)	21 (30)	0.066

Multivariate analysis included proteins found to be significant (ER, PTHrPR1, OPN_cyt_) or of borderline significance (CaSR and CK5/6) in univariate analysis. Of those, conserved ER and absent OPN_cyt_ proved to be independently associated with the increased occurrence of BM (*p* = 0.002 and *p* = 0.018, respectively).

**Fig. 3:**
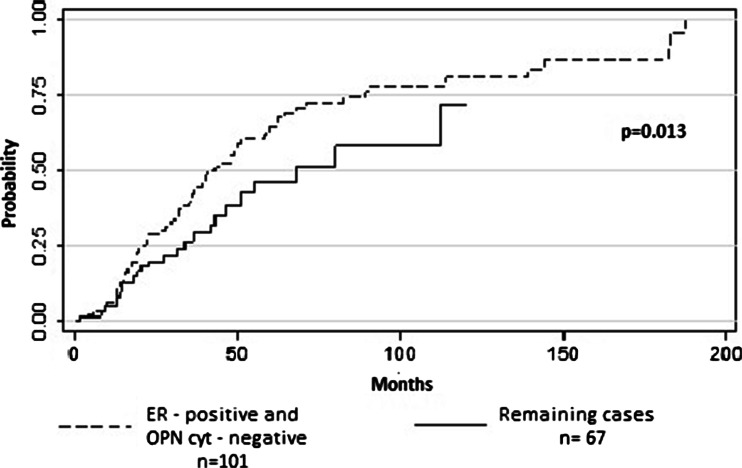
Cumulative incidence of bone metastases in patients with ER-positive/OPN_cyt_-negative immunohistochemical signature vs. others. Excluded were 16 patients in whom the assessment of either ER or OPN staining was not possible

The ER-positive/OPN_cyt_-negative phenotype was significantly more common in BM compared to non-BM group (75 and 25 %, *p* < 0.0001, respectively; HR 1.79 [95 % CI 1.09–2.72]; *p* = 0.013, Fig. 3).

Intrinsic molecular breast cancer surrogates could be determined in 160 subjects (87 %; Table 4). In the BM group the most common subtype was luminal A (43 %), compared to 23 % in the non-BM group (*p* = 0.009). Luminal B/HER2-negative subtype constituted 15 and 21 % of cases in both groups, respectively (*p* = 0.32), and luminal B/HER2–positive subtype - 16 and 4.9 %, respectively (*p* = 0.032). There was no significant difference in the occurrence of nonluminal/HER2-positive subtype between both study groups (9.1 vs. 13 %, respectively, *p* = 0.42). Triple negative subtype was less common among patients with- compared to those without BM (16 and 38 %, respectively, *p* = 0.002).

**Table 4:** Breast cancer phenotypes in patients with – and without BM (significant p values marked in bold)

Phenotype	Bone metastases group n (%)	Extraskeletal metastases group n (%)	*p* value
Luminal A	43 (43)	14 (23)	**0.009**
Luminal B HER2 (−)	15 (15)	13 (21)	0.32
Luminal B HER2 (+)	16 (16)	3 (4.9)	**0.032**
Nonluminal HER2 (+)	9 (9.1)	8 (13)	0.42
Triple negative	16 (16)	23 (38)	**0.002**

## Discussion

Numerous studies investigated the predictive role of potential novel biomarkers of BM in breast cancer, but their clinical utility has not been established [12, 24, 25]. Our study showed that expression of three proteins: ER, OPN_cyt_ and PTHrPR1 in primary breast cancers may be associated with increased risk of BM. We also confirmed apparently different occurrence of BM in particular intrinsic breast cancer surrogates. In concordance with other studies [6, 7], ER positivity was associated with relatively high occurrence and increased risk of BM. For PgR the trend was similar, but the difference did not reach statistical significance. The data on the association between PgR positivity and the occurrence of BM are inconsistent [7].

In our study CaSR was expressed in the vast majority of patients and trended to be more pronounced in patients with BM. Preclinical data suggest that CaSR stimulation can trigger synthesis and secretion of PTHrP through EGFR pathway [26]. PTHrP is also a key player in pathogenesis of osteolytic metastases. Clinical data on the role of CaSR in the development of BM in breast cancer patients are scarce, however similar results were published by Mihai et al. [16]. Despite some differences between both studies, they provide a strong signal of potential CaSR role in pathogenesis of BM.

We hypothesized that high occurrence of BM might also be associated with expression of OPN_cyt_. OPN promotes osteolysis by enabling adhesion of osteoclasts to the bone matrix, and stimulates angiogenesis [14]. Animal studies suggested that breast cancer cells expressing OPN have higher prevalence to BM, particularly if they coexpress IL-11, another potent osteolytic factor [24]. Other studies showed that OPN stimulates progression of cancer and distant metastases [27], and is an adverse prognostic factor [15]. We found significantly lower occurrence of OPN_cyt_ in patients with BM. To our knowledge this is the first report on such relationship. However, owing to small number of cases staining for OPN_cyt_, our results should be interpreted cautiously. Apart from cytoplasmic staining, we also demonstrated less typical nuclear expression of OPN. The biological role of such occurrence is not well understood. Some studies demonstrated increased nuclear content of this protein during cell division in S phase, suggesting its role as a transcriptor factor involved in cell proliferation [28].

In this series PTHrPR1 expression was less common in patients with- compared to those without BM. Physiology of PTHrPR1 is less understood than that of the corresponding peptide (PTHrP). It is possible, that auto- and/or paracrine stimulation of the receptor can lead to synthesis and secretion of PTHrP, which in turn stimulates osteolysis. As mentioned earlier, the whole process can be mediated by CaSR and EGFR. A retrospective study by Hoey et al. [13] showed that expression of PTHrPR1 is more common in BM compared to primary tumors. It was therefore postulated that expression of PTHrPR may be related to preponderance to BM. On the other hand, some data indicate that PTHrPR expression can be stimulated by the microenvironment of the metastatic cancer tissue [13]. Expression of PTHrPR in primary breast cancer was shown to be an adverse prognostic factor [12]. Indeed, PTHrPR stimulation was postulated to promote cell proliferation, invasiveness [29] and angiogenesis [30].

Taken together, OPN and PTHrPR1 play an important role in pathogenesis of BM in breast cancer patients but they are also potent molecular factors involved in cancer progression per se. This could potentially explain higher occurrence of both proteins in primary tumors of patients with extraskeletal metastases, where survival was worse.

Higher occurrence of ER–positivity and OPN_cyt_–negativity in tumors that formed BM was confirmed in the multivariate analysis. Notably, patients with ER–positive/OPN_cyt_–negative phenotype had particularly high risk of BM. If confirmed in further studies, this finding may prompt new preventive and therapeutic strategies.

Consistent with other studies [5, 7], luminal A subtype was found to be the most common phenotype in patients with BM. Additionally, also luminal B/HER2-positive subtype was more common in the BM group. Kennecke et al. [5] estimated that the 15-year cumulative risk of BM in this latter subtype is around 30 %, i.e., higher than in triple negative and luminal A subtypes (16 and 18 %, respectively). Our results should be treated with caution however, given a small number of luminal B/HER2-positive cases. As expected, the extraskeletal metastases (mostly visceral) were most common in the triple negative subset of patients.

## Conclusion

Our study showed that ER expression and the absence of OPN_cyt_ expression are strong and independent factors predicting increased risk of BM in breast cancer patients. Additionally, BM occurrence was specifically associated with luminal A and luminal/B HER2-positive subtypes.

We are aware that our study has several limitations. The methods of IHC staining for investigational proteins may be subjective, vary between particular studies and are not standardized. For most of the proteins we used previously developed scoring systems. The exceptions were CaSR and PTHrPR1, for which we constructed our own scoring systems, partially based on available literature. The obvious limitation of our study is its retrospective nature. In consequence, the non-BM group might have included patients with clinically occult BM, missed in routine management. Further, BM diagnosis was determined using diagnostic methods with different sensitivity (X-ray, CT, MRI, bone scintigraphy). Hence, independent validation of our results is warranted to consider their potential clinical relevance.
